# Louisville Medical Club

**Published:** 1855-07

**Authors:** John Bartlett

**Affiliations:** Secretary


### Art. III.—LOUISVILLE MEDICAL CLUB.

The Louisville Medical Club met at the house of Dr. Rogers on Tuesday evening, June 5.

President Ewing in the chair, and the following members present: Gross, Richardson, Palmer, Rogers, D. W. Yandell, Raphael, Wible, Thomson, Knight, Marshall, J. Bartlett.

Dr. Rogers being called upon for his facts in relation to the scurvy, as lately prevalent here, said that he had himself observed a number of cases, and that he had learned of others upon inquiry. There was no doubt but that the disease observed was identical in nature with sea-scurvy. The cases were characterized by the following symptoms: dingy skin, bloated face, petechiæ and ecchymosis, particularly on the lower extremities, in fact almost confined to them—in one case only there was ecchymosis on the face—muscular soreness, mistakenly treated for rheumatism, pain in lower extremities, causing great suffering; in some cases pain in the chest, though this, contrary to the statement of writers, was not so common as the pain in the legs; some patients suffering with the pain in the chest had been treated for pleurisy, pneumonia, and some had been cupped for the pains in the legs. Most of these patients were confined to bed, and those that could walk complained of stiffness in the knees. In all the cases sponginess of the gums was a well-marked symptom. Both gums were swollen and had a fungous appearance; they were ulcerated, and the teeth were loose; there was occasional hemorrhage. In one of the cases of ulcerated gums there was caries of the alveolar process—no ulcerations existed elsewhere. The tongue and teeth were foul. There was in no case profuse hemorrhage but in some a constant bleeding; bleeding from the nose common—occasionally it occurred from the bowels, and in one or two cases there was hemorrhage from the uterus. In no case was there bleeding from the lungs or bladder.

The diagnosis was plain. It occurred among the poor, and almost exclusively among the Germans or Irish. He had instituted particular inquiry as to whether any case occurred among Americans. Not one case was observed among them. The disease was equally common to both sexes. All of Dr. Rogers' cases were women; the most of Dr. O'Reilley's were men. Many children were affected, and no age was exempt. The disease appeared most in the suburbs, but frequently in the middle portions of town. It has prevailed in Jeffersonville, New Albany, and Portland. In some cases the disease manifested itself as far back as January, in others it commenced in March and April. Every patient had, beyond a doubt, been deprived of vegetable food, especially of potatoes, during the winter. Most of the patients had lived upon fresh meat and bread. The Germans had not their usual supply of krout. Some of the Irish patients had lived almost exclusively on corn bread.

The mortality has been very slight; no noncomplicated case has died. A few cases in children, complicated with hooping-cough, died. A number of the worst cases supervened on intermittents, and all so complicated were bad.

The treatment was rather dietetic than medicinal. The patients recovered rapidly upon vegetable food, especially potatoes, and lemon juice. In fact, lemon juice or citric acid is as much a specific for scurvy as quinine and sulphur are specifics for intermittent fever and scabies. The cure in all cases was prompt and rapid, requiring only a few weeks—even in one week an amendment was very perceptible. For the pains no special means were used.

To some questions of Dr. D. W. Yandell's, Dr. Rogers here answered, that he was not certain whether the stiffness of the limbs continued after the subsidence of the lividity of the gums—he thinks it did. The bowels were rather constipated. He did not see any children at the breast affected. There were four or five children at the Alms House with it. The appetite was always good, and vegetables agreed with the patients; acids were agreeable to them.

Dr. D. W. Yandell stated that in thirty of his cases, twenty-seven had obstinately constipated bowels; twenty said they had chills, and in many instances the stiffness of the limbs continued for some length of time after other symptoms had disappeared.

Dr. Wible was requested to prepare a paper on cholera infantum for the next meeting.

Dr. Richardson's invitation to meet at his house on the next evening was accepted, and the club adjourned.

JOHN BARTLETT, *Secretary.*

---

*The Louisville Medical Club* met at the house of Dr. T. G. Richardson, Tuesday evening, July 3. President Ewing in the chair, and the following gentlemen present: Drs. Gross, Richardson, Yandell, Knight, Bemis, Wible, Bartlett, and D. W. Yandell. Dr. W. M. Yandell, of Mississippi, was introduced to the club.

The paper of the evening was read by Dr. Wible, on *cholera infantum:*

GENTLEMEN OF THE LOUISVILLE MEDICAL CLUB:—When you appointed me to make a report upon the intestinal affections of children, I suppose it was intended that I should give an account of what was prevalent of these diseases during the interval of our meeting. I have, however, thought that this kind of a report would prove tedious to you, particularly as I have no new facts to offer; and, at any rate, I prefer to avail myself of this opportunity to present some thoughts upon the nature and treatment of cholera infantum; and I hope that the members, if they cannot agree with me in my views of the disease, will, at least, grant indulgence for the liberty I have taken.

In the large cities of the United States, cholera infantum is among the most frequent and fatal of the affections of children It is more so than in the cities of Europe. The cause of this probably is owing to the greater and more prolonged heat of summer, and to the negligence of sanitary regulations. A defective treatment may have a relation with the mortality; and the subject to us is one that deserves our best attention.

Dr. Eberle is of opinion, that an essential element of the disease consists in an erethism of the brain, brought about by the process of dentition, and that this erethism is analagous to inflammation; or, to use his language, to "the morbid condition which constitutes, what is ordinarily called acute hydrocephalus;" that this cerebral irritation being reflected upon the stomach and bowels, renders them preternaturally irritable, and in this condition, the child being enfeebled by the heat of summer, "the slightest reduction of temperature, a current of fresh air, or damp night air, will cause a torpor" of the cutaneous emunctories; "the blood will retreat from the surface to the internal organs, and give rise to engorgement of the vessels of the liver, and mucous membrane of the bowels, by which the gastro-intestinal irritability will be still further increased and the characteristic symptoms of the disease excited." He regards the vomiting and purging, the convulsions and coma as produced by the cerebral, irritation. In none of our standard authorities upon the diseases of children, have I seen the nervous element of cholera infantum presented in any very different light. The doctrine, however, is not in accordance, with what we know of the functions of the nervous system. The experiments of physiologists show that *irritation* of the brain produces no such effects as vomiting and purging; and further, the error is one calculated to mislead in practice. It leads to the use of blood-letting and revulsives; and we find that Eberle had recourse to these identical measures, to relieve what he supposed to be a morbid condition of the brain.

It is true that nervous irritation is a prominent feature in the disease, and one of its essential elements. The vomiting, the muscular twitching, the starting in sleep, and from noise, are features not to be overlooked. This nervous excitability is owing, not to any morbid condition of the brain, but to *irritation of the peripheral extremities of the incidental spinal nerves;* the various causes of the irritation acting according to laws well understood and admitted by all physiologists.

The causes of cholera infantum acting slowly in this manner, finally set up an *excitable* or *impressible* state of the spinal centers, and when this is established the irritating causes act with still greater force. We have something analagous to this in traumatic tetanus, where the persistence of a local irritation induces an intensely excitable state of the spinal centers, so that the slightest irritation, of even a distant part from the original, will throw the whole muscular system into spasmodic action. Analagous states of the spinal centers are induced by a great variety of causes. In hysteria, and puerperal convulsions, this state precedes the muscular disturbance; and is induced, in the former by uterine and intestinal irritation, and in the latter, by various causes incidental to pregnancy.

This erethitic condition was described by Darwin as "increased sensorial power;" or "increase of the spirit of animation," and more recently by Dr. Marshall Hall, as augmented excitability of the vis nervosa. That it is the spinal centers affected, is capable of the clearest demonstration, the laws and modes of action of that part of the nevous system now being well understood. The erethism which we find in the cholera infantum, is undoubtedly an affection of the same division of the nevous system; and I find, since I commenced writing this paper, that Dr. Tyler Smith adopts what I have attempted to show, as a fact, and he proceeds to make use of it in illustration of another subject. He says: "Place the nipple between the gums of the infant, and it excites the movement of suction and deglution, and nothing more. Let the same gums be irritated continually by the advancing teeth, and instead of producing at once a reflex action of any kind, the irritation slowly excites the whole system to such a pitch of intensity that every nerve and muscle connected with the system is after a while engaged in successive fits of violent convulsions." "The entire system is, so to speak, loaded or saturated with excitability." "And so long as the alveolar irritation continues, the excitability or morbid augmentation of the vis nervosa increases; while, if the local disorder be removed, the excitability, the charged state of the stomach, diminishes." To present fully the nervous element in cholera infantum, all the laws, both physiological and pathological of the spinal system ought to be given; but this of course cannot be done on an occasion like the present. It will be sufficient to state that this part of the nervous system is called into activity, especially by irritation and the emotions.

The irritations which develop the erethism, or augmented excitability, displayed in cholera infantum, are—

1. Dentition, irritating the nerves distributed to the gums.
2. Summer heat, irritating the nerves distributed to the skin.
3. Impure air, especially that of large cities, and ill-ventilated apartments, irritating the nerves distributed to the lungs.

4. Accumulations in the alimentary canal, irritating the nerves distributed to the stomach and intestines.

A combination of these causes is required to develop the disease; no *one* of them being sufficient. When the augmented excitability becomes induced, the irritations and emotions act with morbid force upon the motor system, producing disorder of functions.

The distinction between ordinary vomiting and purging in children, and cholera infantum, consists in this, that the fomer supervenes upon a normal state of the nervous functions, and the latter upon augmented excitability.

The following is the connection of pathological events in the disease:

1. Several causes acting upon the peripheral extremities of the spinal incident nerves, produce, slowly, an erethism or state of augmented excitability of the spinal centers or the spinal system.

2. The same causes, or new ones, or the emotions acting upon this increased irritability, produce a pathological state of the motor apparatus.

3. Abdominal action of the motor apparatus produces congestion and disorder of function.

4. Congestion and derangement of function lead to organic changes, such as inflammation of the mucous follicles of the intestines, enlargement of the liver, &c.

During the progress of the disease the morbid secretions and the inflammation produced, become sources of irritatoin, and add further to the gravity of the malady, and from this we may infer the importance of *early* attention to the treatment.

It will not be disputed, I suppose, that the irritations and the emotions acting on the spinal system, will produce disorder and disease.

The indications for treatment, according to the principles I have attempted to present, are as follows:

1. To remove every source of irritation.
2. To reduce the augmented excitability.
3. To *prevent* exhaustion.
4. To obviate congestion and inflammation.
5. To sustain the strength.

*First:* To fulfill the first of these indications, the apartment of the child should be airy and quiet, the clothing loose, clean and easily changed, and if the locality be insalubrious, the child should be removed to a healthy place. An advancing tooth ought to be liberated at an early period by free and repeated scarifications. The stomach and bowels, if they contain undigested food, or offensive and irritating secretions, ought to be thoroughly evacuated. A grain each of rhubarb, bicarbonate of potassa, and carbonate of magnesi, given several times a day, constitutes about the best purgative.

*Second:* To overcome the augmented excitability, the removal of the various sources of irritation is, of course, of the first importance. Besides this much may be done by the judicious use of the warm bath, and by hyoscyamus. The former soothes, and the latter absolutely controls the excitability. The warm bath should be repeated from time to time when there is much restlessness or other nervous symptoms. I usually give from the fourth to a half of a grain of the extract of hyoscyamus every two or three hours, until the pupils become dilated, or the nervous symptoms are subdued. I almost always combine this medicine in the form of an emulsion, with two or three grains of the bicarbonate of potassa, for the purpose of keeping the stomach free from acidity.

*Third*: To prevent exhaustion, which will arise from profuse purging, astringents have often to be early and promptly employed. The acetate of lead, tannin, some of the vegetable astringents, or the chalk mixture, may be selected. It must be remembered that the tendency to exhaustion is very great, and requires the watchful care of the physician.

*Fourth:* To remedy local congestion and inflammation is the most difficult part of the treatment. The removal of irritation and morbid excitability constitutes the first and most important step. The use of minute doses of calomel, opium, and ipecacuanha, is in some cases of much service; but the utmost discrimination must be made in the use of these medicines. The calomel, by its purgative effects and by its exciting irritating secretions, may tend to hasten exhaustion and to keep up the abnormal excitability. In children, in certain states of the nervous system, the use of opium is dangerous; sometimes it increases the excitability, and this effect may be ascertained by observing the contracted pupils, and the irritability of the muscular system generally. When the pupils become contracted, or the retching and vomiting increased, or any tendency to convulsions is apparent, the use of opium must be instantly discontinued; otherwise convulsions, coma, or an aggravation of the disease generally, and even fatal effects may be produced.

When inflammatory action in the intestines is severe, leeches to the abdomen, or to the anus, may sometimes be employed with benefit; the tendency, however, to exhaustion, and consequently to increase of nervous excitability, must not be lost sight of while employing them. Fomentations to the abdomen, and tepid mucilaginous injection into the bowels, are useful adjuvants in treating the inflammation.

For the principles which I have suggested it will be inferred that sinapisms blisters and rubefacients could not fail to be injurious. The cardinal points in the treatment, however, are the removal of irritants and the subduing of excitability, and if these are properly attended to there will be little use for remedies to combat inflammation; but, unfortunately, the advice of the physician is not sought or heeded, in many cases, until the secondary effects of the disease have been produced.

*Fifth:* The sudden or gradual exhaustion which we often find must be met by the judicious employment of diet, tonics, and stimulants. The cerebral disturbances which indicate exhaustion in the disease, must *not* be treated by revulsives and calomel, but by those means which soothe and support the system.

It will be perceived that I have advised no new remedies; and if the affection will be seen from the point of view which I have attempted to present, those remedies with which we are already acquainted may be more scientifically and successfully applied. And, finally, if we study the spinal nervous system—the great centinel of the animal frame, the system that sends every morbid impression which it receives to the entire organism, and the agent which distributes the innumerable sympathies that we find in disease—we will have a fuller comprehension not only of cholera infantum, but of almost every other disease.

Most of the members took part in the discussion which followed.

Dr. Yandell said that cholera infantum was much less prevalent now than when he commenced practiee twenty-odd years ago, and in the last ten years especially, according to his observation, there had been a marked abatement in both its extent and intensity. He thought it much more amenable to treatment than formerly. Twenty-five or thirty years ago the disease was not cured with the facility that it is now. Patients would relapse—continue sick until frost, in spite of medicine. He now usually succeeded in curing the affection with calomel, ice, chalk mixture and opium. Chalk julep, with a few grains of the submuriate were generally sufficient in ordinary cases. One of the most interesting facts in regard to cholera infantum, was its occasional periodicity. Fifteen years ago he saw a case in which this feature was well marked. He treated the paroxysm by cold water, and in the interval gave quinine. Recovery was prompt. He has since had frequent occasions to pursue the same course. He made a publication in the Western Journal some years since in regard to the connection which sometimes exists between malaria and cholera infantum. He did not agree with those authors who held that the affection under consideration was a disease of large cities. He thought it by no means confined to cities. It prevailed extensively in Tennessee and Kentucky, and was especially rife in miasmatic districts.

Dr. W. B. Caldwell agreed with Dr. Yandell in regard to cholera infantum being by no means confined to cities. He had seen more of it in the six years he had practiced in the country, in the interior of Kentucky, than he had done in Louisville in nine years. In the country it was expected that every child would suffer from it during its second summer, and the expectation was generally realized. His observation led him to the opinion that the disease was most common in miasmatic districts, and not unfrequently assumed an intermitting character. When this was the case he had been in the habit of using anti-periodics with good results. He regarded the disease as more manageable here than in the country.

Dr. Ewing thought there was no question that the disease was n many instances connected with malaria. He regarded it as much less prevalent in Louisville now than it was twenty years ago, and as decidedly more tractable. He had used quinine in cases adapted to the remedy with great satisfaction. He had understood that the disease was both less frequent and severe in Nashville since that city had been supplied with river water, but supposed it a mere coincidence, as the same thing did not hold good in other places which were similarly supplied.

Dr. Gross remarked that while cholera infantum was confessedly both less violent and frequent in Louisville than in either Lexington, Ky., Cincinnati, or St. Louis, he did not think the cause of the difference was so apparent. During his residence in Cincinnati, extending over a period of seven years, it was an exceedingly common and fatal disorder. He had seen no violent cases for eighteen months. He had repeatedly seen a form of cholera infantum not mentioned generally by authors which was excessively rapid and fatal, highly inflammatory, accompanied by the most distressing thirst, jactitation, and a manifest tendency to convulsions. During his practice in Cincinnati a case came into his hands which died in twenty-two hours from the time it was attacked. He had known other instances in which death occurred in even half that time. He saw no reason for thinking that the use of hydrant water diminished in any way the disposition to cholera infantum. Hydrant water was used at Cincinnati, St. Louis, and Philadelphia, and there could be no question that the disease was much more common and fatal in those cities than here, where we used a very strong limestone water. In regard to the opinion expressed by members as to the greater tractability of the affection compared to what it was a qurter of a century ago, he would ask if it could not be legitimately ascribed to its better management. He had met with but few cases in which quinine was indicated.

Dr. Yandell did not regard the treatment of cholera infantum as differing materially from what it was twenty-five years ago. He was willing to admit that the improved dietetic and hygienic management of the disease might have something to do with its diminished fatality, but his observation was that as a disease cholera infantum was much less frequent than formerly. Bilious fever, he believed, had undergone the same changes. He thought it far less frequent now than it was twenty years ago. Both bilious fever and cholera infantum have evidently decreased since the advent of cholera in 1833. He thought it an established fact that endemic diseases disappeared before epidemic diseases, that as the latter prevailed the former disappeared. The average mortality was about the same. The deficiency created by the absence of one class of affections was compensated by the presence of the other.

Dr. Rogers could not speak from personal observation as to either the extent or mortality of cholera infantum twenty-five years ago. He thought it a sufficiently common disease now. He saw a great number of cases every summer, and many of them were fatal. In the months of June and July he thought it of very general prevalence, and often really exceedingly unmanageable. He had seen cases of the character alluded to by Dr. Gross—had known children to die in an incredibly short time. They seemed to be fatally diseased from the very start. Acting upon the suggestion of Dr. Yandell, contained in his article on cholera infantum, he had used quinine, but could not say it had yielded in his hands what was claimed for it by other members. He had met with cases in which it was required, and in which its good effects were decided and immediate, but such cases were very rare. He was not in the habit of trusting much to mercurials, in fact he seldom gave them, and never unless the dejections were light-colored and thin; he preferred hydr. c. cret. He seldom gave calomel. In the majority of cases he thought the chalk mixture and tr. opium succeeded better than any other formula. He also used the acids and mineral astringents. He does not think well of the acetate of lead.

Dr. Wible begged leave to differ with Dr. Rogers. In his hands acetate of lead fulfilled an excellent purpose. He was in the habit of using it in most cases which required an astringent. He gave no opium. A favorite prescription with him was one grain each of powdered rhubarb, bicarb. potash and carb. magnesia. He had used it in a great number of cases with entire satisfaction. He exhibited acetate of lead sometimes in almost heroic doses. He gave to a child not long ago a half grain of the acet. plumb. every hour for twelve hours with the best results. He very rarely gives calomel.

Dr. Knight remarked that he should dislike exceedingly to be obliged to treat cholera infantum without mercurials. He does not administer much opium. When he finds the stomach quiet he gives calomel, chalk mixture, and kino. He agreed with those members who regarded the disease as less frequent and more amenable to treatment than formerly. He entertains also the same opinion in regard to bilious fever.

Dr. W. M. Yandell, of Mississippi, does not think cholera infantum either so violent or common in Mississippi as he found it twenty years ago, when he practiced in Middle Tennessee. Still, there is more or less of it almost every summer in Mississippi. He thinks that in many instances its connection with miasma can be clearly traced. He used both quinine, calomel, chalk mixture, and opium. He thought that this disease was not an exception to other diseases as they prevailed in the South, and that in most instances quinine was demanded.

Dr. D. W. Yandell had been governed a good deal in the management of cholera infantum by the indications laid down by a German physician, whose name he had forgotten. Where the discharges were dark, green, or sour, he trusted to alkalies exhibited in strong mint tea. Where, on the other hand, the dejections were light-colored, he relied upon a dose or two of calomel, and then gave chalk mixture and tr. opium. Where there was much febrile disturbance he used cold water bath, the acids and ice. He had seen cases relieved almost at once by quinine. In properly selected cases he regarded this salt as one of the most valuable of remedies. He had used it that day with a child who lived in the lower part of the city with the most satisfactory results. He had been called to visit a child once in Tennessee whose case was regarded as hopeless. A simple dose of quinine arrested the vomiting, and in a day it was convalescent. He might cite other instances. He not unfrequently found a change of air succeed after all medicine had failed. He had known children exhausted by this disease to be relieved by a trip to Cincinnati. During his sojourn in the country for two summers in Tennessee, he had seen but little of the complaint, and thought that in the particular district in which he had lived it was neither a very frequent nor obstinate disease.

Dr. Gross proposed that members should report at the meeting to be held in January next, whether morning sickness is or is not present in the majority of cases of pregnancy falling under their observation.

Dr. Richardson proposed that members examine all cases of hooping-cough that may present themselves between this and the next regular meeting, and report at that time relative to the ulceration on the frenun linguæ, which is said to be one of the features of the affection. He himself had seen but one case of hooping-cough, and in that the ulcer was present.

The meeting then adjourned to meet on the first Tuesday in August at the house of Dr. Yandell.

JOHN BARTLETT, *Secretary.*

